# Facile synthesis of multi-faceted, biomimetic and cross-protective nanoparticle-based vaccines for drug-resistant *Shigella*: a flexible platform technology

**DOI:** 10.1186/s12951-023-01780-y

**Published:** 2023-01-29

**Authors:** Namrata Baruah, Nadim Ahamad, Prolay Halder, Hemanta Koley, Dhirendra S. Katti

**Affiliations:** 1 Department of Biological Sciences and Bioengineering, Indian Institute of Technology Kanpur, Kanpur, 208016 Uttar Pradesh India; 2 The Mehta Family Centre for Engineering in Medicine, Indian Institute of Technology Kanpur, Kanpur, 208016 Uttar Pradesh India; 3 Division of Bacteriology, ICMR-National Institute of Cholera and Enteric Diseases, Kolkata, 700010 West Bengal India

**Keywords:** Biomimetic, Nanoparticle, *Shigella*, Nanovaccine, Antibiotic resistance, Shigellosis, Vaccine

## Abstract

**Background:**

No commercial vaccines are available against drug-resistant *Shigella* due to serotype-specific/narrow-range of protection. Nanoparticle-based biomimetic vaccines involving stable, conserved, immunogenic proteins fabricated using facile chemistries can help formulate a translatable cross-protective *Shigella* vaccine. Such systems can also negate cold-chain transportation/storage thus overcoming challenges prevalent in various settings.

**Methods:**

We explored facile development of biomimetic poly (lactide-*co*-glycolide)/PLGA 50:50 based nanovaccines (NVs), encapsulating conserved stabilized antigen(s)/immunostimulant of *S. dysenteriae* 1 origin surface-modified using simple chemistries. All encapsulants (IpaC/IpaB/LPS) and nanoparticles (NPs)—bare and modified (NV), were thoroughly characterized. Effect of IpaC on cellular uptake of NPs was assessed in-vitro. Immunogenicity of the NVs was assessed in-vivo in BALB/c mice by intranasal immunization. Cross-protective efficacy was assessed by intraperitoneally challenging the immunized groups with a high dose of heterologous *S. flexneri* 2a and observing for visible diarrhea, weight loss and survival. Passive-protective ability of the simplest NV was assessed in the 5-day old progeny of vaccinated mice.

**Results:**

All the antigens and immunostimulant to be encapsulated were successfully purified and found to be stable both before and after encapsulation into NPs. The ~ 300 nm sized NPs with a zeta potential of ~ − 25 mV released ~ 60% antigen by 14th day suggesting an appropriate delivery kinetics. The NPs could be successfully surface-modified with IpaC and/or CpG DNA. *In vitro* experiments revealed that the presence of IpaC can significantly increase cellular uptake of NPs. All NVs were found to be cytocompatible and highly immunogenic. Antibodies in sera of NV-immunized mice could recognize heterologous *Shigella*. Immunized sera also showed high antibody and cytokine response. The immunized groups were protected from diarrhea and weight loss with ~ 70–80% survival upon heterologous *Shigella* challenge. The simplest NV showed ~ 88% survival in neonates.

**Conclusions:**

Facile formulation of biomimetic NVs can result in significant cross-protection. Further, passive protection in neonates suggest that parental immunization could protect infants, the most vulnerable group in context of *Shigella* infection. Non-invasive route of vaccination can also lead to greater patient compliance making it amenable for mass-immunization. Overall, our work contributes towards a yet to be reported platform technology for facile development of cross-protective *Shigella* vaccines.

**Graphical Abstract:**

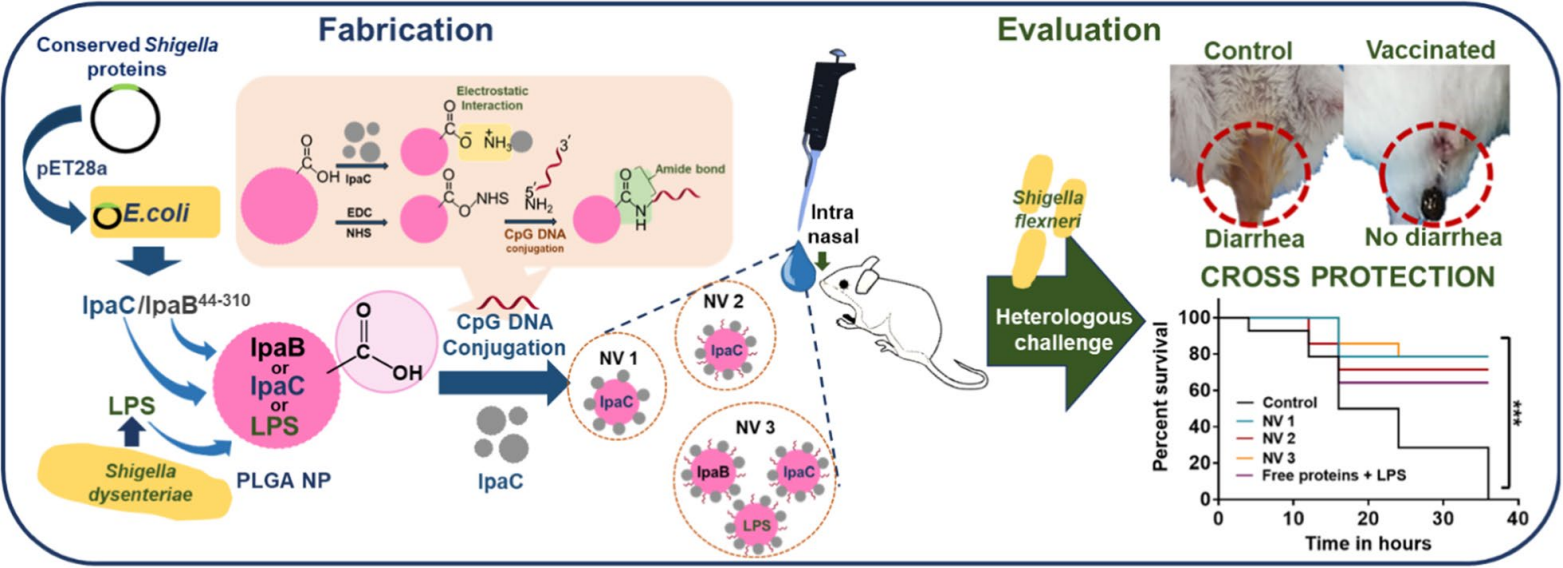

**Supplementary Information:**

The online version contains supplementary material available at 10.1186/s12951-023-01780-y.

## Introduction

Globally, the leading cause of bacterial diarrhea and the second leading cause of diarrheal deaths is *Shigella*, a group of Gram-negative bacteria responsible for > 0.2 million deaths in 2016 [1]. It causes the disease shigellosis symptoms of which are abdominal cramps, tenesmus, diarrhea (bloody), fever, vomiting and nausea [2]. Associated complications range from severe dehydration, toxic megacolon, hemolytic uremic syndrome, rectal prolapse, seizures (especially in children) to sepsis and death in neonates and malnourished children with long-term repercussions like stunted physical growth and reactive arthritis [2, 3]. Infectious diarrhea can severely affect the elderly and children, especially infants with a low fluid volume, cumulatively resulting in millions of deaths per year [4, 5]. Effects of the disease are more damaging in parts of the world with lack of access to clean drinking water, hygiene, nutrition and healthcare [1].

Although, multiple strains of *Shigella* have developed drug resistance and recent major outbreaks are caused by resistant *Shigella*, unfortunately, a commercial vaccine is yet to be available [2, 6–14]. Hence, the requirement for a *Shigella* vaccine is paramount.

Since, *Shigella* has four species—*S. dysenteriae* (caused maximum epidemics/pandemics in the previous century, results in a severe form of the disease), *S. flexneri* (currently, most common globally), *S. boydii* and *S. sonnei* (second to *S. flexneri* in occurrence) with more than 50 serotypes and subserotypes, development of a vaccine protecting against all *Shigella* is a challenging task [2, 15–19]. Therefore, despite considerable efforts towards development of a translatable vaccine, several long-standing challenges including low immunogenicity and/or serotype-specific narrow-range protection impede progress [20, 21]. In this context, conserved antigens common to all *Shigella* are of particular interest for providing cross-protection.

All *Shigella* infect host cells using a needle-like structure referred to as the Type III secretion system (T3SS) [22]. A few proteins of this system known as the invasion plasmid antigens (Ipa) such as IpaB, IpaC and IpaD (available at the tip of T3SS during host cell infection) have shown cross-protective ability against heterologous *Shigella* in their recombinant forms [23–26]. These recombinant proteins are of significance as they do not involve pathogenic *Shigella* in the purification process which not only reduces cost but also significantly reduces exposure to pathogenic *Shigella* during large-scale manufacture. A complex of Ipa proteins IpaB and IpaC along with lipopolysaccharide (LPS) (Invaplex) has been previously shown to have significant protective ability and is currently under clinical trials [27]. However, instability of recombinant Ipa proteins and requirement of continuous cold-chain transportation and storage raise major hurdles for facile and rapid development of a translatable vaccine [28, 29]. This prompted us to stabilize recombinant IpaC (*S. dysenteriae* 1-most unstable IpaC) at various temperatures including room temperature in our previous report which provided cross-protection against heterologous *Shigella* challenge [26].

Further, polymeric particulate vaccine systems could be explored since encapsulation in a particle matrix protects the antigens from pre-mature degradation [30, 31]. Additionally, slow and sustained controllable release and efficient uptake in mucosal-associated lymphoid tissues (MALT) result in minimization of dosage and long-lasting immune activation [30, 32–34]. Further, delivering multiple antigens and immunostimulants can enhance immune response at a minimal dose [35]. Hence, herein, we explored encapsulation of stabilized recombinant IpaC and immunodominant region of recombinant IpaB (IpaB^44-310^) as antigens and LPS as an immunostimulant into polymeric delivery systems which were amenable to lyophilization enabling room temperature stability/transportation.

Choice of the polymer significantly affects antigen release kinetics and in turn, immune activation [30, 36]. The biocompatible and biodegradable poly (lactide-*co*-glycolide) or PLGA is one of the most widely explored safe (FDA approved for a few drug delivery applications) [37] polymers for drug/antigen delivery systems with known adjuvanticity [38–44]. However, surprisingly, it has limited exploration for *Shigella* vaccine development wherein only a handful of studies involving nanoparticulate-delivery systems have been reported [45–48]. Size of such polymeric systems can also significantly affect cellular uptake and immune cell activation. PLGA systems of ~ 300 nm is reported to result in high efficacy [49].

Additionally, strategic surface modification of particles by mimicking biological aspects of pathogenesis can result in greater vaccine efficacy [40, 50–53]. Immunostimulants like CpG DNA (synthetic oligodeoxynucleotides containing CpG motif frequently present in bacterial genome), considered as pathogen-associated molecular patterns (PAMPs), are widely used in biomimetics for their ability to generate robust antibody titers and increased Th1 response when added to protein vaccines [40, 54–57]. As surface modification/biomimicking using CpG DNA increases vaccine efficacy of nanoparticles, we conjugated CpG DNA on the surface of PLGA nanoparticles [55, 58, 59].

Further, as increasing cellular uptake can reduce vaccine dosage [60], *Shigella* Ipa proteins such as IpaC which play a vital role for pathogen entry (part of T3SS) by polymerizing actin, can be explored as a biomimetic strategy to facilitate the process of cellular uptake and consequential efficiency of the nanovaccines [34, 61–63].

Non-invasiveness is another desirable factor for translatable vaccines as it results in higher patient compliance with potential for mass immunization [64]. While challenges like pre-mature antigen degradation associated with oral vaccines can be partially overcome through the use of polymeric delivery systems, the intranasal route of immunization provides higher protection from pre-mature degradation due to the lack of an acidic environment [65]. Intranasal delivery also leads to lesser accumulation in vital organs like liver and faster absorption owing to a large absorption surface [66]. Due to the common mucosal immune system, intranasal immunization can result in distant site-specific immunity (including the intestinal site, relevant for shigellosis) along with enhanced mucosal as well as systemic immunity [65, 67–69].

Therefore, herein, we describe PLGA nanoparticle-based intranasal vaccines encapsulating *Shigella* antigens and immunostimulant with increasing level of complexity [nanovaccines (NV)—NV1, NV2 and NV3] to arrive at an appropriate vaccine. The encapsulants were conserved recombinant *Shigella* antigens IpaB^44-310^ or IpaC or the immunostimulant LPS of *S. dysenteriae* 1 (Sd1) origin. These antigen/immunostimulant loaded nanoparticles were surface modified with CpG DNA and/or IpaC using facile chemistries to obtain biomimetic nanovaccines. The immunized mice were intraperitoneally challenged with a high dose of heterologous *S. flexneri* 2a (Sf2a) and observed for visible diarrhea and weight loss. Additionally, the possibility of passive protection was also explored.

Overall, the current work is the first report to describe a facile platform technology for the rapid development of non-invasive and cross-protective biomimetic nanovaccines for shigellosis which minimizes the requirement of cold-chain transportation and storage.

## Materials and methods

### Bacterial strains

*Escherichia coli *cells DH5α and BL21 (DE3) Rosetta were obtained from Microbial Type Culture Collection, India and cultured in Luria Bertani (LB) broth/agar and nutrient broth/agar (HiMedia, India). *Shigella flexneri* 2a (B294) (Sf2a) and *Shigella dysenteriae* 1 (NT407) (Sd1) were obtained from National Institute of Cholera and Enteric Diseases, India, maintained in a biosafety level 2 facility and cultured in tryptic soy broth/tryptic soy agar/Hektoen enteric agar (Difco, USA).

### Extraction and purification of encapsulants

LPS was extracted from *Shigella dysenteriae* 1 as previously reported [70] and its quality and quantity was evaluated using silver staining and thiobarbituric acid assay (TBA assay) respectively. The standard used for TBA assay was commercial *Shigella* LPS procured when it was available from Sigma Aldrich, USA. All chemicals used in the process were of analytical grade obtained from HiMedia (India) Sigma Aldrich (USA), Merck (India) and Loba Chemie (India). Due care was taken while handling *Shigella*. The purified LPS was lyophilized and stored at − 20 °C.

Recombinant Sd1 IpaC was purified as previously reported [26]. The stable size exclusion chromatography-purified protein was evaluated using SDS-PAGE, circular dichroism spectroscopy (Jasco J-815 CD spectrometer) and western blotting with anti-mouse anti-histidine tagged primary antibody and goat-anti-mouse HRP-labelled secondary antibody (Sigma Aldrich, USA). The immunodominant region of IpaB (IpaB^44-310^) was expressed in *E. coli* as previously reported [26, 71], purified by affinity and size exclusion chromatography and evaluated as described for IpaC. Both IpaC and IpaB^44-310^ were quantified using BCA assay kit (Merck, India).

### Formulation of nanovaccines

The purified LPS, IpaB^44-310^ and IpaC were separately encapsulated in biodegradable polymeric nanoparticles (NPs) developed using carboxylic acid-terminated poly (lactide-*co*-glycolide) or PLGA 50:50 co-polymer with 0.55–0.75 dL/gm inherent viscosity (Lactel Absorbable Polymers, USA). The NPs were prepared using a previously reported double emulsion solvent evaporation method with slight modifications (Additional file 1: Fig S1) [72–74]. Briefly, LPS/IpaB^44-310^/IpaC in 0.025% polyvinyl alcohol (PVA) (Sigma Aldrich) was gradually introduced in 2.5% w/v PLGA solution prepared in dichloromethane (DCM, Merck). The resulting solution was sonicated for 1 min at 40% amplitude to make a primary emulsion which was introduced drop wise in 2% w/v aqueous PVA solution under continuous stirring conditions and further sonicated for 3 min at 60% amplitude to obtain the secondary emulsion. It was then continuously stirred for ~ 10 h to allow for complete evaporation of dichloromethane, the organic solvent. The NPs were then centrifuged at 12,000 rpm for 20 min at 4 °C. The obtained NP pellet was washed for 6 times using sterile milli-Q water to remove the residual PVA. The purified NPs were then resuspended in sterile milli-Q water and lyophilized for 24 h. Blank PLGA NPs were also prepared similarly, except for the difference of adding milliQ water in place of protein/LPS.

### TRITC conjugation of protein and formulation of fluorescent NPs

IpaB^44-310^ protein solution was buffer exchanged to 0.1 M sodium carbonate buffer at a pH of 9. TRITC, Tetramethylrhodamine isothiocyanate (Sigma Aldrich, USA) at a concentration of 1 mg/ml in Dimethyl sulfoxide, DMSO (Sigma Aldrich, USA) was prepared and 50 µg of TRITC/2 mg of protein was added. The reaction was performed for 2.5 h with mild shaking in dark conditions. The solution was dialysed into PBS to remove unconjugated dye. The fluorescence of the protein solution was quantified at multiple time points in a microplate reader (BioTek Instruments, USA) to confirm stability of fluorescence. The TRITC-tagged IpaB^44-310^ protein was loaded into PLGA nanoparticles as described earlier while maintaining dark conditions and lyophilized for 24 h.

### Characterization of nanovaccines

The developed nanovaccines were analyzed for hydrodynamic diameter and zeta potential using Zetasizer ZS90 (Malvern Instruments Ltd., UK). The morphology of the particles were studied using electron microscopy (EVO 18, ZEISS and JSM-7100F, JEOL). Further, to evaluate the structural integrity of the encapsulants, released LPS/IpaC/IpaB^44-310^ were examined with gel electrophoresis after dissolving the particles. LPS-NPs in ultrapure water were dissolved using dichloromethane and the aqueous fraction obtained post centrifugation was silver stained. IpaC/IpaB^44-310^ encapsulated NPs were dissolved in 0.1 M NaOH solution containing 5.0% w/v SDS in a rotary shaker, as reported previously [43]. After 2 h of incubation, the solution was centrifuged and the supernatant containing the released IpaC/IpaB^44-310^ was analyzed by SDS-PAGE. Additionally, the amount of released LPS was quantified as follows. 5 mg of LPS encapsulated NPs were hydrolysed in 0.2 N H_2_SO_4_ at 100 °C for 30 min. Free LPS was also similarly treated. After cooling for 7 min, the tubes were centrifuged at 14,000 rpm for 5 min. The supernatants were collected for performing TBA assay. IpaC/IpaB^44-310^ in the supernatant of dissolved particles (as described above, 24 h of shaking) was quantified using BCA assay post centrifugation. The percentage loading and percentage encapsulation efficiency were calculated using the equations provided below.

$$\mathrm{\% }Loading=Amount \, of \, encapsulant \, per \, mg \, of \, dry \, NPs \times 100$$

$$\mathrm{\% }Encapsulation \, efficiency=\frac{Amount \, encapsulated \, \left(mg\right) \, in \, NPs}{Amount \, of \, encapsulant \, used \, for \, loading \, (mg)}\times 100$$

For assessing the release kinetics, IpaB^44-310^ loaded NPs (as IpaB^44-310^ has longer durability at 37 °C compared to IpaC) were incubated in PBS at 37 °C under shaking conditions (pH 7.4) for 14 days. The supernatant was collected at fixed intervals to quantify the amount of released antigen using micro BCA assay.

### Surface modification with CpG DNA

The acid-terminated PLGA nanoparticles were functionalized with 1% 5′ amine-terminated CpG DNA 1826 (5′-TCCATGACGTTCCTGACGTT-3′) on the basis of EDC-NHS chemistry. Briefly, 25 mg/ml of bare nanoparticles were taken and equal volumes of 0.4 M N-(3-Dimethylaminopropyl)-N′-ethylcarbodiimide hydrochloride (Sigma Aldrich, USA) and 0.1 M *N*-Hydroxysuccinimide (Sigma Aldrich, USA) were added. After 1 h of gentle shaking at room temperature, 1% CpG DNA (Integrated DNA Technologies, USA) was added. 2 h post shaking at room temperature, the particles were centrifuged at 12,000 rpm for 15 min. The nanoparticle pellets were washed for 3 times, resuspended in ultrapure water and lyophilized for 24 h. The percentage of CpG modification was indirectly calculated from the supernatant and the subsequent washings with the help of qualitative agarose gel electrophoresis and quantification of the band intensities using ImageJ software [75]. Quantification was also performed using NanoDrop 2000 (Thermo Fisher Scientific, USA). Surface modification of the PLGA NPs was also detected using Attenuated Total Reflection-Fourier Transformed Infrared (ATR-FTIR) spectroscopy performed on Bruker Tensor 27 IR spectrometer (Bruker Optik, Germany) equipped with an ATR accessory. Mercury cadmium telluride (MCT) was used as the detector.

### Cytocompatibility

The cytocompatibility of the nanovaccines were assessed on human colorectal epithelial adenocarcinoma cell line, CaCo2, procured from ATCC^®^ HTB-37™. The cells were cultured in Dulbecco’s Modified Eagle’s medium (DMEM) supplemented with 10% v/v fetal bovine serum and 1% v/v penicillin–streptomycin solution. Around 9 × 10^3^ cells were seeded in each well of 96-well plate and maintained at 37 °C and 5% CO_2_. The cells were treated with media containing NPs (20 µg/ml) and a separate group of cells was treated with blank NPs. After 24 h of incubation, cytocompatibility in the treated groups were evaluated using LDH cytotoxicity assay kit (Thermo Scientific, USA) according to manufacturer’s instructions.

### Nanoparticle uptake

2 × 10^5^ cells of human colorectal epithelial adenocarcinoma cell line (CaCo2) were seeded on each cover slips placed inside 6-well plates and allowed to adhere for 1 h. 2 ml DMEM/well was added without disturbing the cover slips and incubated for 12 h. 1 ml of DMEM was then replaced with sterile 1× PBS and 100 µl of 0.5 mg/ml of the respective groups of fluorescent nanoparticles were added to the wells. In both the IpaC treated groups, 100 µg/ml of IpaC (lyophilized, resuspended) was added along with the fluorescent nanoparticles with or without CpG DNA modification. Another group was incubated with only the fluorescent NPs without CpG DNA modification. After 30 min of incubation of the NPs, the media was removed and the cover slips were washed with PBS and prepared for confocal microscopy imaging using Zeiss LSM 710.

### Animals

Vaccination studies were conducted on 4–6 week old BALB/c mice of either gender. The animals were housed in sterile cages maintained at 25 ± 2 °C and 65 ± 2% humidity with timely provision of sterile food and water. All animal experiments were conducted according to the protocol approved by the Institutional Animal Ethics Committee (IITK/IAEC/2016/1042) both at IIT Kanpur and NICED, Kolkata.

### Immunization

Lyophilized nanovaccines were reconstituted in PBS and used for intranasal immunization in BALB/c mice (n = 14/group determined using power analysis) on 1st, 14th and 28th day. The treatment groups were as follows- NPs encapsulating 10 µg of IpaC, CpG DNA modified NPs encapsulating 10 µg of IpaC, mixed NPs separately encapsulating 10 µg IpaC, 40 µg IpaB^44-310^ and 2.5 µg LPS, all of which were surface modified with CpG DNA. All these NPs were administered in a solution of 10 µg of free IpaC in PBS (lyophilized, resuspended at the time of immunization). Therefore, all the groups had equal amount of total IpaC. Total CpG DNA in both NV2 and NV3 was also constant. Additionally, two control groups were included viz., free form of the encapsulants (IpaB^44-310^, IpaC and LPS, the stabilized IpaC and extracted LPS were lyophilized and resuspended at the time of study) and Control/blank nanoparticles.

### Immunoblotting and ELISA

Immunoblotting was performed to assess the ability of generated antibodies in sera of immunized mice to recognize both homogenous and heterologous *Shigella* spp. For this, overnight cultures of Sd1 and Sf2a were centrifuged and the cell pellets were resuspended in PBS and lysed by heating at 95 °C for 5 min in the presence of SDS. The lysates were resolved on 12% SDS-PAGE and transferred onto nitrocellulose membrane. Sera obtained at 28th day were used as the source of primary antibody while HRP-tagged antibodies were used as the secondary antibody. The immunoblots were assessed using chemiluminescence. Generation of the antibody response was evaluated in sera of immunized mice on 1st, 14th, 28th and 45th day of primary immunization. For this, an overnight culture of Sd1 was centrifuged and the cell pellet was resuspended in PBS containing 0.05% w/v LDAO which was then sonicated for 10 min to generate whole cell lysate (WCL). 100 µL WCL was coated in each well of 96-well ELISA plate and allowed to incubate overnight at 4 °C. Sera stored at – 20 °C were used as a source of primary antibody (lowest dilution—1:50) and HRP-tagged antibodies were used as the secondary antibody. The HRP chromogenic substrate o-phenylene di-amine (OPD) in 0.1 M citrate buffer (pH 4.5) with H_2_O_2_ was used for color development. The reaction was stopped with 2 N H_2_SO_4_ and final absorbance was measured at 492 nm. IL-6, IL-10 and IFN-γ cytokine levels in 35th day sera of immunized mice was quantified using cytokine ELISA assay kits (Invitrogen, USA) as per manufacturer’s instructions.

### Antibiogram

Antibiogram of the challenge organism *S. flexneri* 2a B294 was performed using Kirby-Bauer disc diffusion method. For this, 400 μl of log-phase culture of *Shigella* in LB broth was spread on Mueller Hilton Agar plates. The antibiotic impregnated discs (HiMedia, BD Biosciences) were then placed on the agar plates and incubated at 37 °C overnight. The zone of inhibition (mm) of each plate was measured the next day and analysed using data sheets provided by the manufacturers.

### Shigella challenge

The protective efficacy of the nanovaccines were evaluated by challenging the treated groups with heterologous *S. flexneri* 2a. The mice were intraperitoneally injected with 1 × 10^9^ CFU of Sf2a/mouse on the 56th day of primary immunization. The challenged animals were continuously monitored for overall health conditions and the onset of visible diarrheal symptoms 4 h post challenge. The percentage survival of the treated groups was recorded till 14 days post-challenge.

### Passive protection

The immunized adult mice were bred to obtain progeny. The obtained 5-day old neonates were orally challenged with 5 × 10^8^ CFU of Sf2a/mouse based on a prior bacterial dose determination study. A control group of 5-day old neonates (parents vaccinated with blank NPs) were simultaneously challenged for comparison. The percentage survival was recorded till 40 h of challenge.

### Statistical analysis

All the experiments were performed at least in triplicate (n = 3). The results are presented as mean ± SEM unless otherwise mentioned. GraphPad prism 6 software package was used for calculations. Statistical difference in results was analyzed using two-way analysis of variance, one-way analysis of variance, unpaired *t*-test and Log rank’s test wherever applicable.

## Results and discussion

### Extraction and purification of encapsulants

**Fig. 1:**
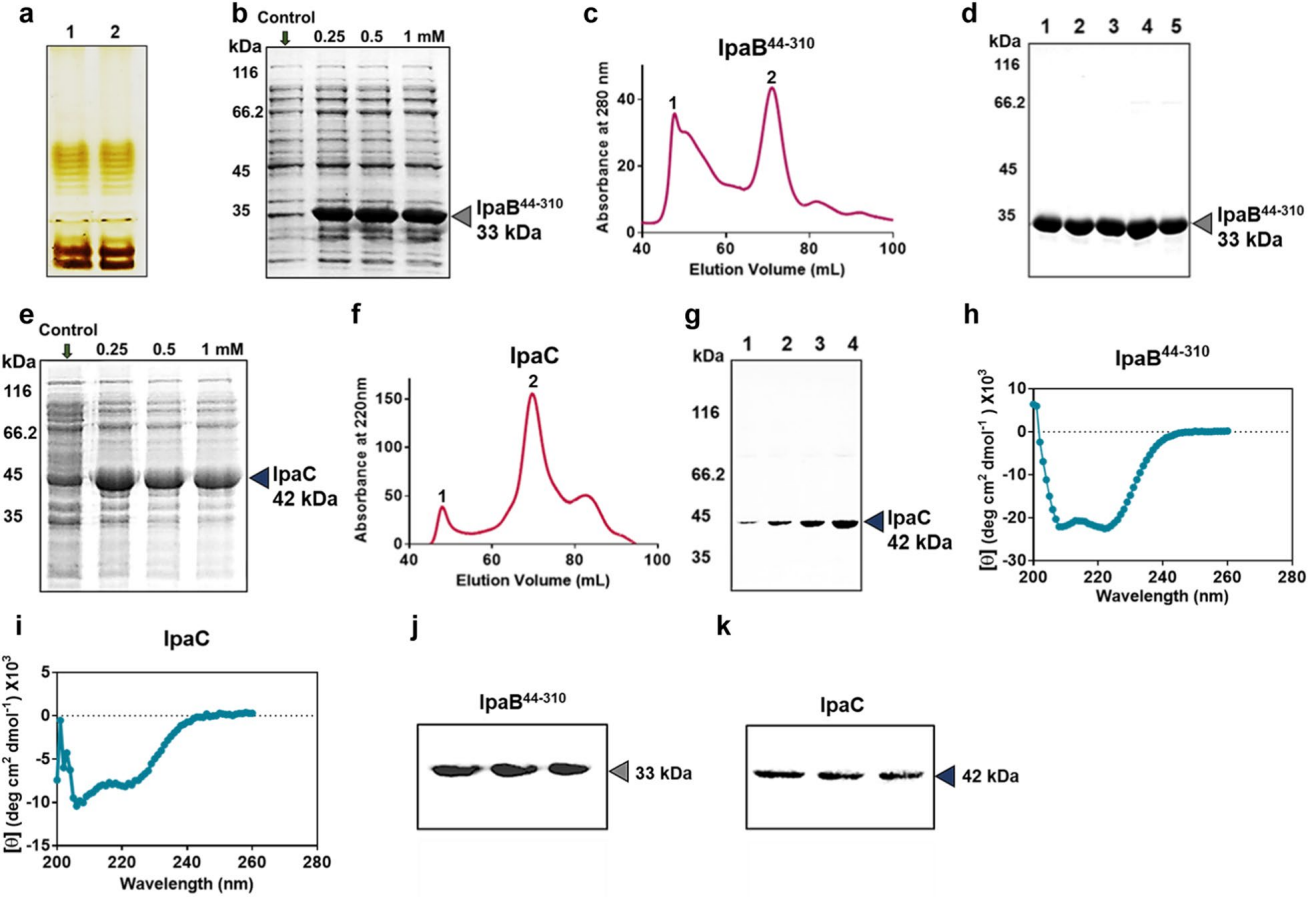
Extraction and purification of LPS, IpaB^44-310^ and IpaC. **a** Silver staining of LPS extracted from Sd1 (lane 1) and Sf2a (lane 2). **b**–**d** Characterization of IpaB^44-310^—**b** SDS PAGE of expressed IpaB^44-310^. Lanes represent bacterial cell lysates of uninduced control and IPTG induced groups (0.25, 0.5 and 1 mM respectively). **c** Size exclusion chromatography profile (SEC). **d** SDS PAGE of a few SEC purified fractions (constant volume) obtained from peak 2 of **c**. **e**–**g** Characterization of IpaC. **e** SDS PAGE of expressed IpaC. Lanes represent bacterial cell lysates of uninduced control and IPTG induced groups (0.25, 0.5 and 1 mM respectively). **f** SEC profile (at 220 nm as Tryptophan is absent). **g** SDS PAGE of a few SEC purified fractions (constant volume) obtained from peak 2 of **f**. **h** and **i** Circular dichroism spectrogram of IpaB^44-310^ and IpaC, respectively. **j** and **k** Western blot of IpaB^44-310^ and IpaC respectively (same fraction obtained from three different batches separately loaded in the three lanes), with anti-mouse anti-histidine tagged primary antibody and goat-anti-mouse HRP-labelled secondary antibody

Sd1 LPS was extracted and characterized via silver staining and thiobarbituric acid assay (TBA) as reported previously (Fig. 1a and Additional file 1: Fig. S2) [70]. A characteristic ladder pattern in silver staining (Fig. 1a) showed the quality of the extracted LPS and pink coloration (Additional file 1: Fig. S2) attributable to keto deoxyoctonate in the LPS backbone, aided the colorimetric quantification of LPS via TBA assay.

IpaB^44-310^ (33 kDa) and IpaC (42 kDa) were expressed in *E.coli* as reported previously [26, 71] and purified by affinity and size exclusion chromatography to obtain highly pure antigens (Fig. 1b–g). Protein fractions eluted in the second peaks (Fig. 1c and f) showed the highest yield (Fig. 1d and g) and for consistency, only these fractions were considered for further experiments (as these membrane proteins frequently tend to oligomerize on gel, faint bands corresponding to dimers were observed). The purified proteins showed characteristic secondary structure profiles upon analysis with circular dichroism spectroscopy (Fig. 1h and i). Prior to loading in nanoparticles, the stored proteins at 4 °C were analyzed by western blotting (Fig. 1j and k). All the extracted encapsulants were stable before loading into NPs.

### Formulation of nanoparticles

**Fig. 2:**
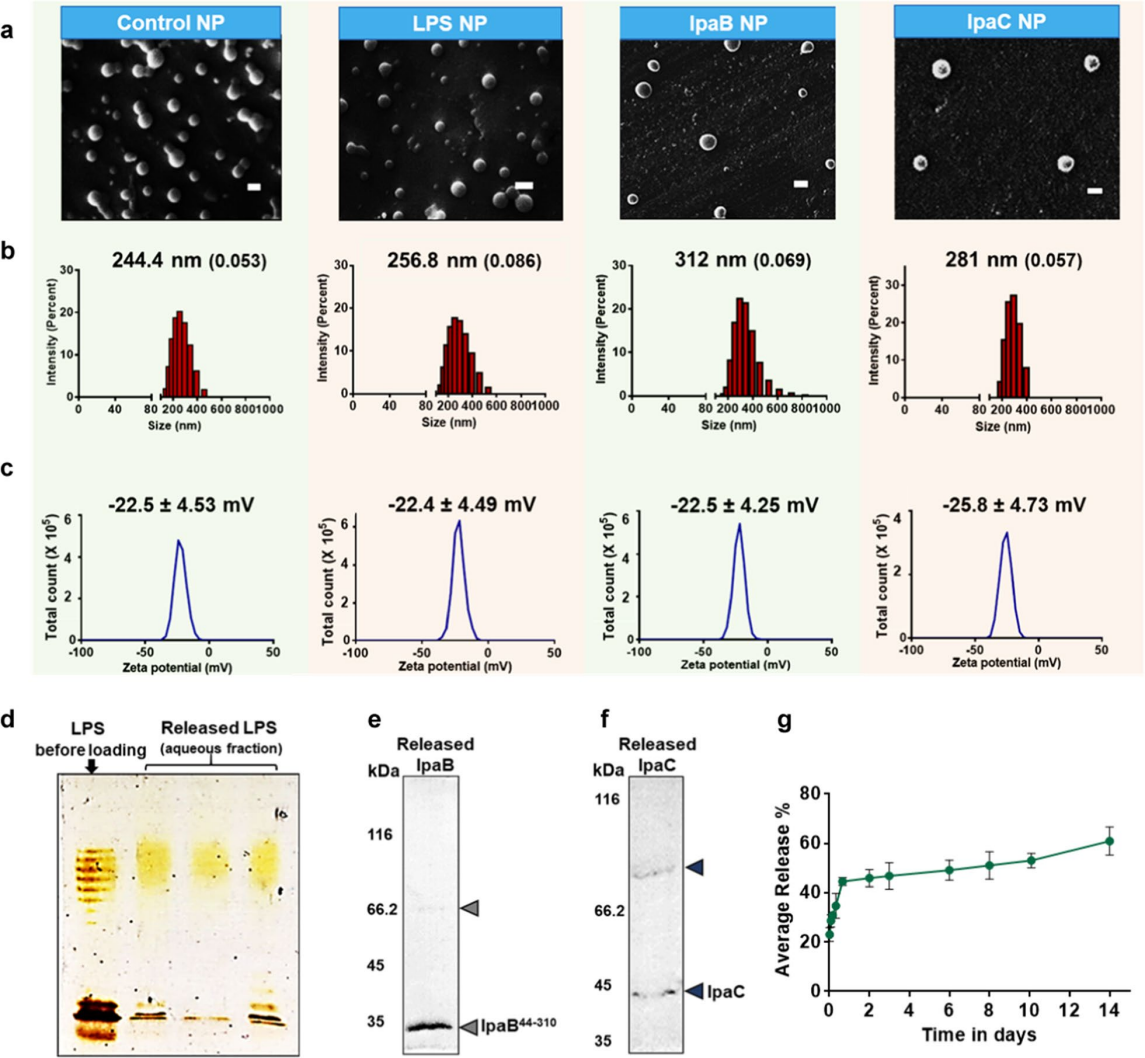
Characterization of nanoparticles (NPs) and released encapsulants.** a** Scanning electron micrographs (scale bar—200 nm). **b** Hydrodynamic size (polydispersity index) and **c** Zeta potential of—Control NPs (Blank), LPS encapsulated NPs, IpaB^44-310^ encapsulated NPs and IpaC encapsulated NPs respectively. The NPs were dissolved and the released encapsulants were analyzed. **d** Silver staining of released LPS. **e** and **f** SDS PAGE of released IpaB^44-310^ and IpaC respectively (arrow heads pointing to bands). **g** Release of model antigen IpaB^44-310^ as a function of time

PLGA nanoparticles (NPs) were then formulated by encapsulating LPS, IpaB^44-310^ and IpaC using double emulsion solvent evaporation method (Additional file 1: Fig. S1). All NPs showed spherical shape with smooth morphology when visualized using scanning electron microscopy (Fig. 2a). Since, preliminary experiments (Additional file 1: Fig. S3) revealed that simultaneous encapsulation of immunostimulant and antigen decreased antigen loading capacity, the encapsulants were loaded in separate NPs. The hydrodynamic diameters of the LPS, IpaB^44-310^ and IpaC encapsulated NPs were approximately 257 nm, 312 nm and 281 nm respectively (Fig. 2b) which were ~ 300 nm and hence, were expected to show high cellular uptake [49]. The zeta potentials of the NPs were − 22.4 ± 4.49 mV, − 22.5 ± 4.25 mV and − 25.8 ± 4.73 mV respectively (Fig. 2c) which allowed for sufficient colloidal stability. Further, in order to understand the possibility of an adverse effect on the encapsulants due to the process of encapsulation, the NPs were dissolved to resolve the released encapsulants on gel (Fig. 2d–f). LPS in the aqueous fraction was analyzed by silver staining (Fig. 2d). Free LPS (prior to loading in NPs) was also treated similarly (first lane). The characteristic banding pattern observed in the aqueous fraction confirmed that the process of encapsulation does not adversely affect the quality of LPS. Upon dissolving the IpaB^44-310^ and IpaC encapsulated NPs and assessing the quality of released protein on gel, distinct bands were observed at 33 kDa (a small percentage in a dimer form at around 66 kDa) (Fig. 2e) and at 42 kDa (dimer at 84 kDa) (Fig. 2f) respectively, suggesting the presence of undegraded protein (15 µg and 0.8 µg respectively). Thus, all the particles prior to surface modification were in the range of 245–312 nm in size with comparable negative zeta potential values (~ − 22 to − 25 mV) and the process of encapsulation did not adversely affect the integrity of the encapsulants.

### Loading and encapsulation in NPs

Table 1 describes percentage loading and percentage encapsulation efficiency in the NPs. LPS released from the dissolved nanoparticles was subjected to TBA assay to obtain an encapsulation efficiency of 16.1% and a loading of 0.4%. The released proteins were assessed by BCA assay to obtain an encapsulation efficiency of 21.7% with loading of 1% for IpaC. On the other hand, IpaB^44-310^ showed relatively higher encapsulation efficiency of 60% with a loading of 3.1%. The release kinetics of the system was studied using IpaB^44-310^ encapsulated NPs as a model system due to relatively higher stability of IpaB^44-310^ at 37 °C when compared to IpaC (Fig. 2g). Approximately 60% of the protein was released by 14th day suggesting release of sufficient antigen for an appropriate immunogenic response.

**Table 1:** Encapsulation and loading of antigen/immunostimulant in NPs

Encapsulants	%Encapsulation efficiency	%Loading	Assay	Amount of encapsulant in NPs used for vaccination (µg/mg NP)
LPS	16.1	0.4	TBA	4.0
IpaC	21.7	1.0	BCA	10.0
IpaB	60.0	3.1	BCA	31.0

### Surface modification and characterization of NPs

**Scheme 1.**
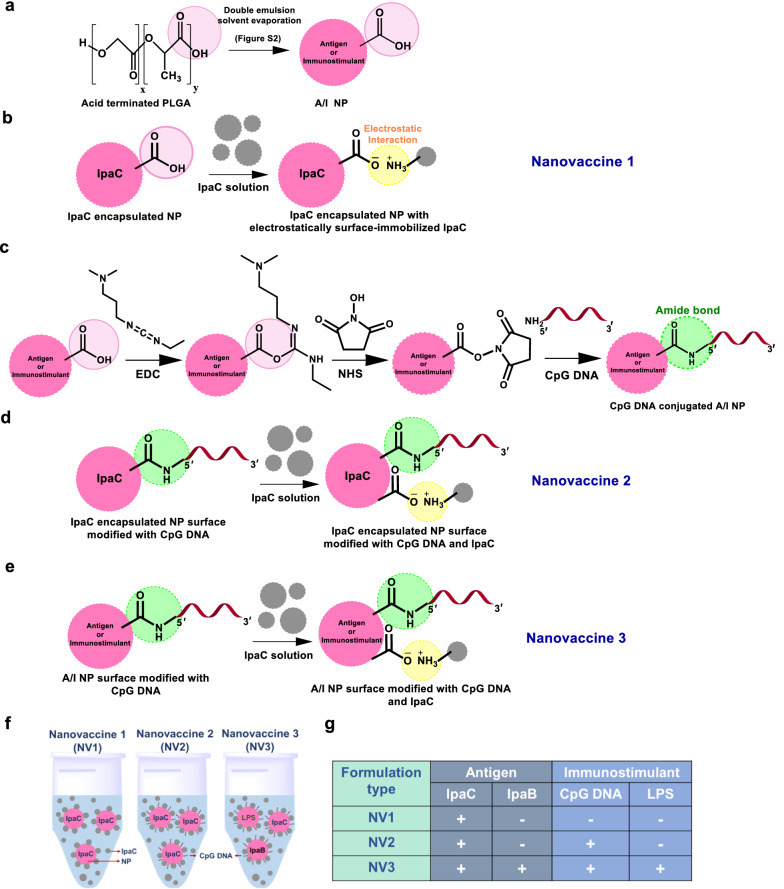
Design of biomimetic nanovaccines. **a** Acid terminated (light pink circle) PLGA was formulated into antigen/immunostimulant loaded nanoparticle (A/I NP—pink circle) encapsulating antigens IpaC or IpaB^44-310^ or an immunostimulant, LPS. **b** IpaC encapsulated nanoparticle suspended in IpaC solution for surface adsorption of IpaC (electrostatic interaction—yellow circle) was termed as Nanovaccine 1 (NV1). **c** A/I NPs surface modified with CpG DNA (covalently linked- green circle) on the basis of EDC-NHS chemistry. **d** IpaC encapsulated nanoparticle, surface modified with CpG DNA, suspended in IpaC solution for surface adsorption of IpaC was termed as Nanovaccine 2 (NV2). **e** All 3 types of A/I NPs surface modified with CpG DNA suspended in IpaC solution for surface adsorption of IpaC, was termed as Nanovaccine 3 (NV3). **f** Pictorial representation of the formulations—NV1, NV2 and NV3. **g** Antigen/Immunostimulant content in each nanovaccine- NV1 has one antigen IpaC, NV2 has one antigen IpaC with one immunostimulant CpG DNA and NV3 involves two antigens IpaB^44-310^ and IpaC along with two immunostimulants CpG DNA and LPS

**Fig. 3:**
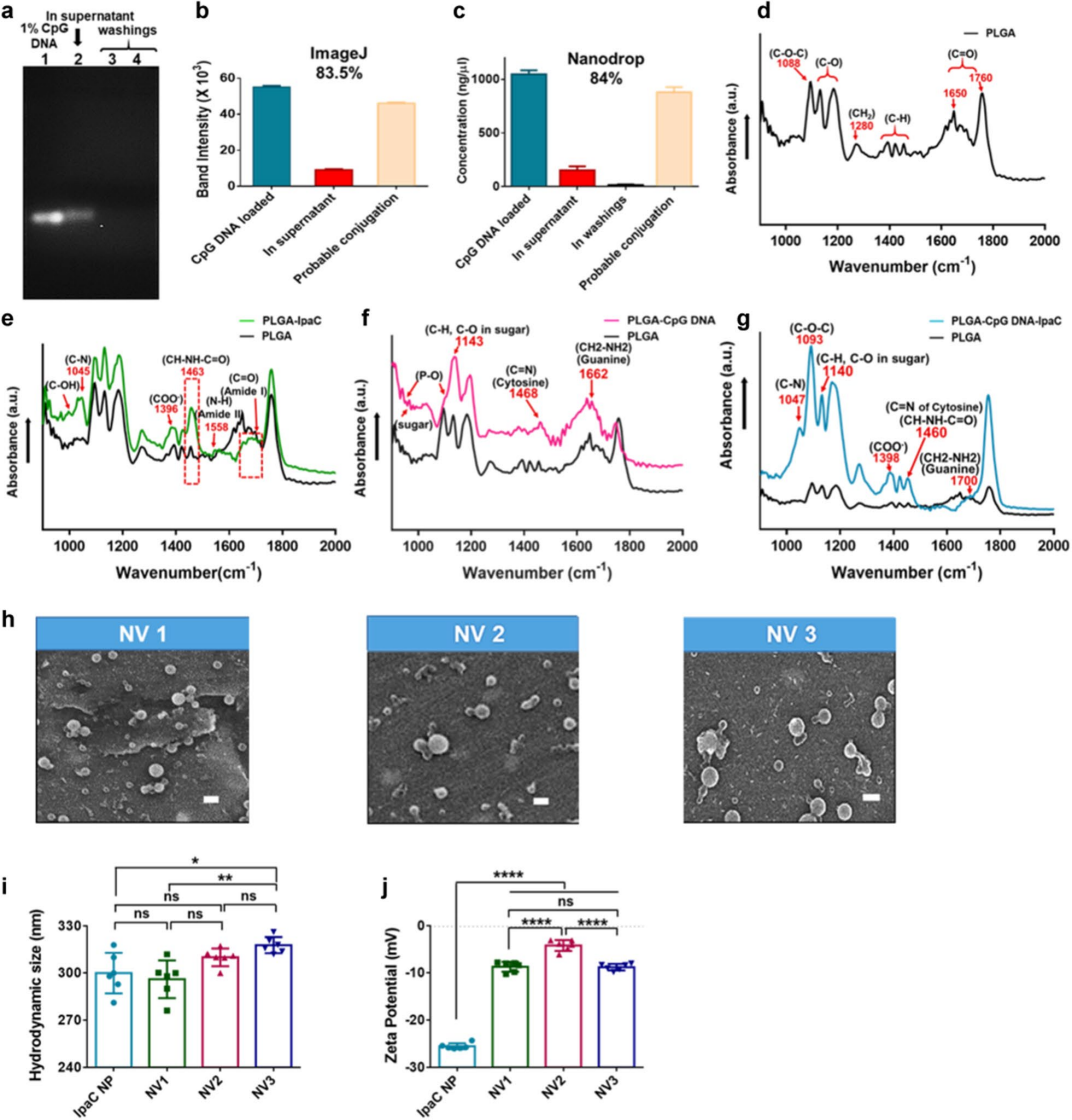
Characterization of surface-modified nanovaccine. **a** Gel electrophoresis of—1% CpG DNA (lane 1), unconjugated CpG DNA in the supernatant (lane 2), washings of consequent steps (lanes 3 and 4). **b** Quantification of DNA band intensities in **a** using ImageJ to obtain probable conjugation. **c** Quantification of probable conjugation using Nanodrop. **d**–**g** ATR-FTIR analysis depicting characteristic/sample-specific peaks for—**d** PLGA NP, **e** PLGA NP in IpaC solution, **f** CpG DNA conjugated PLGA NP and **g** CpG DNA conjugated PLGA NP in IpaC solution. **h** Scanning electron micrographs (scale bar—500 nm), **i** average hydrodynamic size, and **j** average zeta potential of NV1, NV2 and NV3 respectively (n = 6). *p < 0.05, **p < 0.01 and ****p < 0.0001, indicate statistically significant difference between the respective groups; ns indicates non-significance

The biomimetic nanovaccines were designed with increasing degree of complexity (Scheme 1). The nanovaccines were formulated with acid terminated PLGA via double emulsion solvent evaporation method (Scheme 1a). NV1 depicts IpaC encapsulated NPs, suspended in IpaC solution for surface adsorption of IpaC (Scheme 1b, electrostatic interaction). Surface modification of the acid-terminated nanoparticles with CpG DNA was performed on the basis of carbodiimide cross-linker chemistry which results in the formation of an amide bond between carboxy-terminated PLGA and amine-terminated CpG DNA (Scheme 1c, covalent conjugation). IpaC encapsulated NPs, surface–modified with CpG DNA and suspended in IpaC solution was termed as NV2 (Scheme 1d). In NV3, CpG DNA conjugated NPs separately encapsulating IpaC, IpaB^44-310^ and LPS, were suspended in a solution of IpaC (Scheme 1e). The nanoparticles (CpG DNA-modified or unmodified) were suspended in 10 µg of free IpaC solution (lyophilized, resuspended) at the time of immunization (Scheme 1f). A summary of all the immune-active agents of the nanovaccines is provided in Scheme 1g. An indirect quantitative estimation of physically adsorbed IpaC using BCA assay (of the supernatant containing unbound protein) showed ~ 49% and ~ 45% adsorption of IpaC on unmodified and CpG DNA modified NPs respectively (Additional file 1: Fig. S4). The probable CpG DNA conjugation efficiency was qualitatively assessed by resolving the un-conjugated CpG DNA on agarose gel (Fig. 3a, lane 2) and then quantifying the band intensity using ImageJ [75] (Fig. 3b, red bar) which showed a conjugation of ~ 83.52% (Fig. 3b, light orange) by comparing with lane 1 of Fig. 3a (total DNA used for reaction, Fig. 3b, blue bar). Conjugation was also confirmed using Nanodrop by comparing the total loaded DNA (Fig. 3c, blue bar) with the unconjugated DNA in the supernatant (red bar) and washings (black bar) which showed an efficiency of ~ 84% (Fig. 3c, light orange bar). Qualitative characterization of the surface-modified NPs using ATR-FTIR (Fig. 3d–g) showed signature peaks of IpaC protein (green) (Fig. 3e), CpG DNA (purple) (Fig. 3f) and both protein and DNA (cyan) (Fig. 3g). The formulated nanovaccines were then characterized for their morphology (Fig. 3h), hydrodynamic diameter (Fig. 3i) and zeta potential (Fig. 3j). The average sizes of the NVs were largely similar to the bare IpaC nanoparticles prior to surface modification (Fig. 3i). However, the zeta potential values were significantly less negative compared to the unmodified IpaC encapsulated or CpG DNA modified NPs (Fig. 3j and Additional file 1: Fig. S5). Therefore, to improve colloidal stability, the lyophilized NPs [unmodified and CpG DNA modified] were administered in test animals by mixing in IpaC solution in place of surface immobilization with IpaC [as results in decrease in negative charge i.e., reduced stability].

### Cytocompatibility

**Fig. 4:**
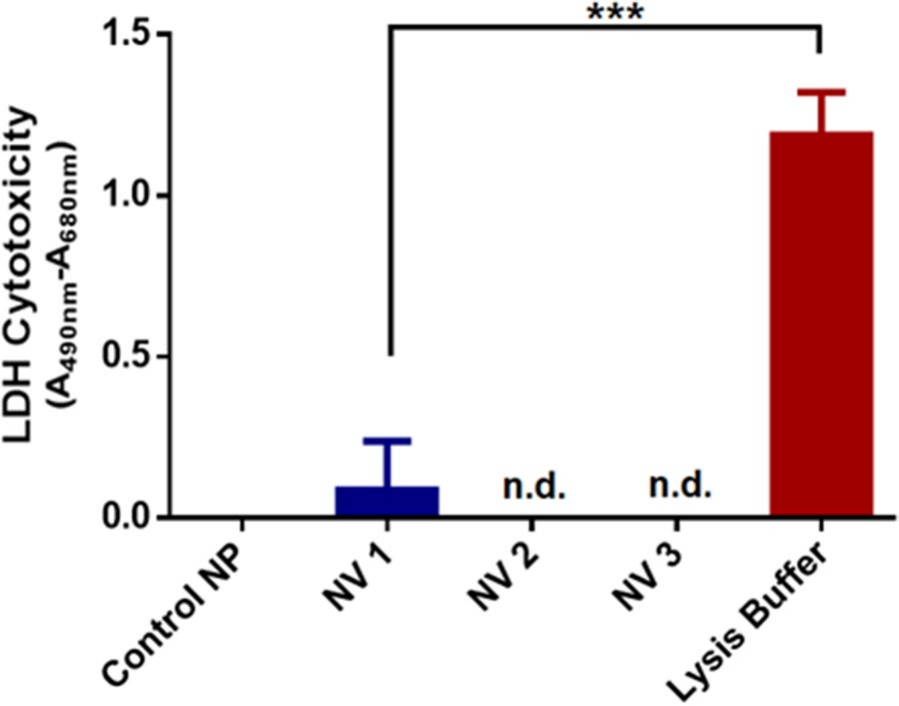
Cytocompatibility of nanovaccines. Quantification of LDH release-based cytotoxicity of the nanovaccines and control NP when compared to lysis buffer (unpaired *t*-test, ***p = 0.0007 with respect to lysis buffer, n.d.—non-detectable)

Cytocompatibility of the nanovaccines was evaluated using lactate dehydrogenase (LDH) activity assay. A higher LDH release/activity indicated a lower cytocompatibility due to loss in membrane integrity of the treated cells (human colorectal epithelial adenocarcinoma cell line, CaCo2). Figure 4 showed that all the nanovaccines (20 µg/ml, concentration that remains in suspension in culture media for at least 24 h) showed minimal or non-detectable LDH release with the response being comparable to the blank/control NPs and hence, were considered cytocompatible.

### Enhanced cellular uptake of NPs in presence of IpaC

**Fig. 5:**
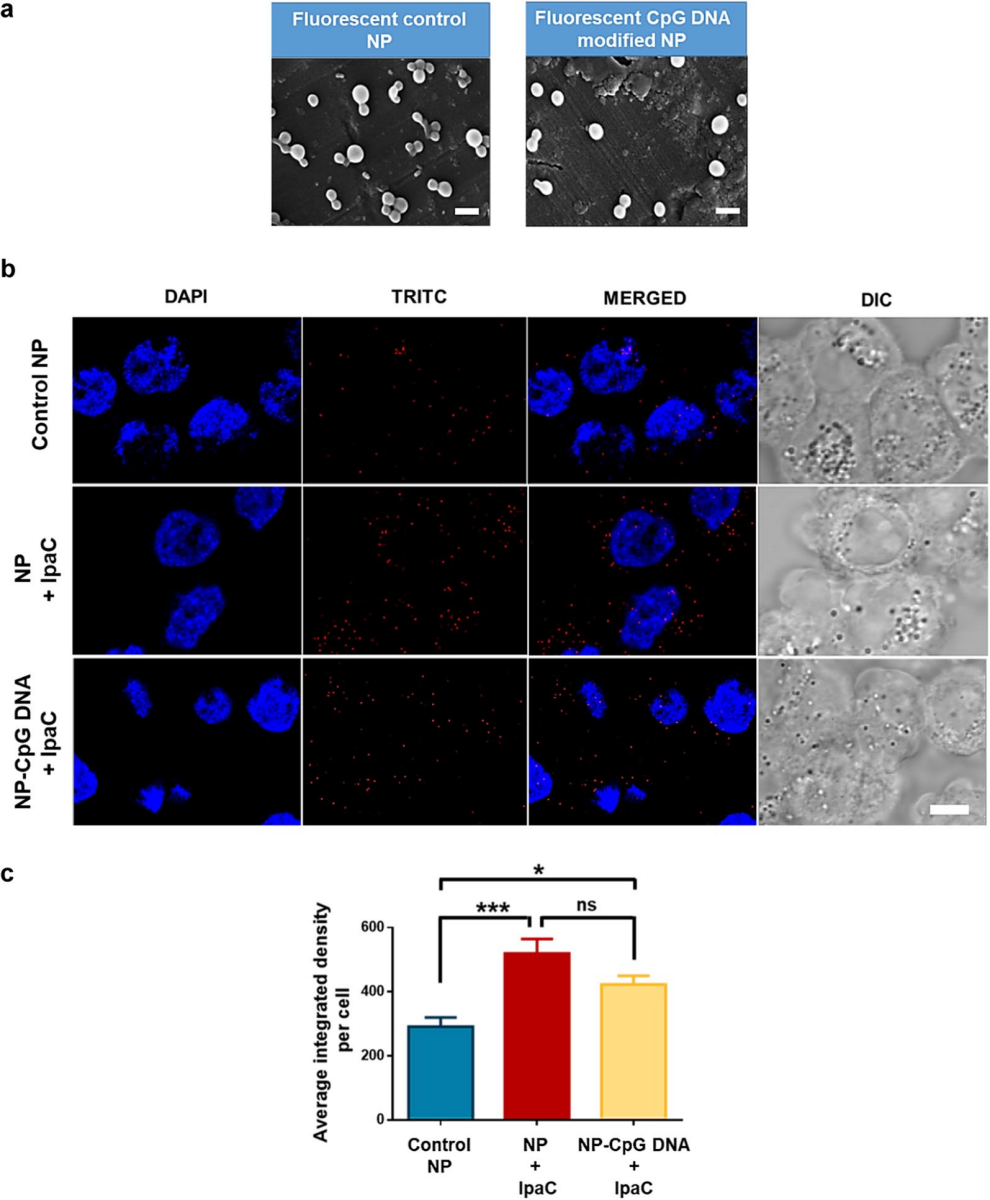
Cellular uptake of nanoparticles in presence of IpaC. **a** FESEM images of fluorescent NPs—control and CpG DNA modified (scale bar—500 nm). **b** Confocal microscopy images of Caco2 cells incubated with nanoparticles [row 1—unmodified control NPs, row 2—unmodified NPs in presence of IpaC (NP + IpaC), row 3—CpG DNA modified NPs in presence of IpaC (NP-CpG DNA + IpaC)]. Blue color depicts DAPI staining of the nuclei and red colour depicts NPs encapsulating TRITC-conjugated IpaB^44-310^ (scale bar—8 µm). **c** Quantification of nanoparticle uptake using Fiji software (One-way ANOVA, ns is non-significant; *p < 0.05 and ***p = 0.0002 indicate statistically significant difference of the IpaC treated groups with respect to control)

To evaluate the effect of IpaC on cellular uptake of NPs, fluorescent NPs encapsulating TRITC-conjugated IpaB^44-310^ (due to its higher loading % and better stability at 37 °C, Fig. 5a, unmodified control and CpG DNA modified NPs) were incubated with Caco2 cells, in presence or absence of stabilized IpaC and examined by confocal microscopy (Fig. 5b). The confocal micrographs showed that compared to the unmodified control NPs in absence of IpaC (Fig. 5b, first row), both unmodified (second row) and CpG DNA modified NPs (third row), in presence of IpaC, showed higher uptake as quantified using Fiji [76] (Fig. 5c). However, the difference in NP uptake between the two groups treated with IpaC (unmodified and CpG DNA modified NPs) was not significant (Fig. 5c). Taken together, the presence of IpaC facilitated the process of NP uptake. Additionally, surface modification of NPs with CpG DNA did not significantly alter the uptake of NPs in presence of IpaC.

### Immunogenicity and cytokine response

**Fig. 6:**
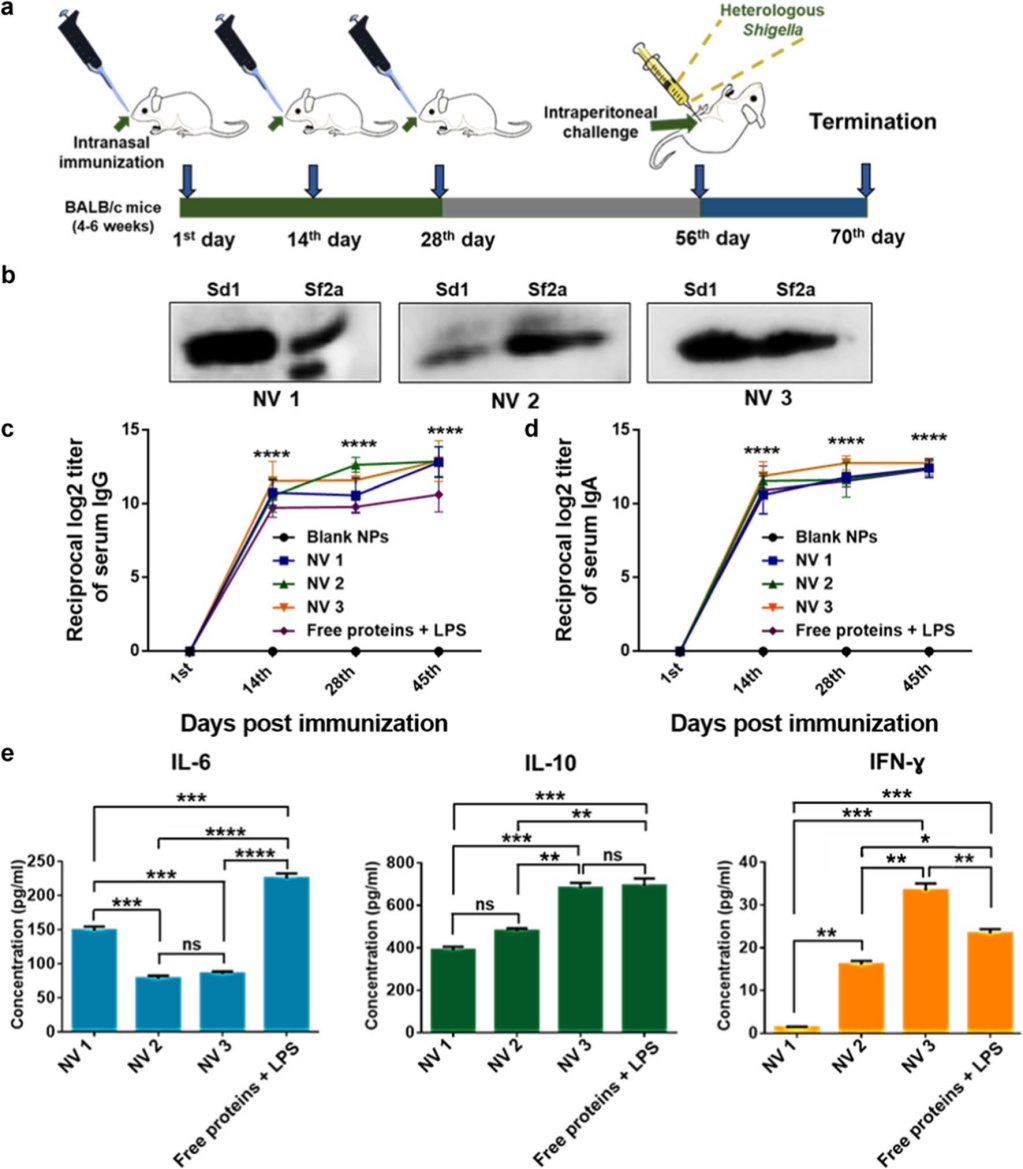
Immunogenicity of nanovaccines. **a** Schedule of immunization. **b** Immunoblot against *S. dysenteriae* 1 (Sd1) and *S. flexneri* 2a (Sf2a) using 28th day sera of nanovaccine immunized mice. **c** Serum IgG and **d** IgA antibody titer assessed by ELISA (n = 3, mean ± S.D.) (two-way ANOVA, Tukey’s multiple comparisons test, ****p < 0.0001 indicate statistically significant difference with respect to control, x-axis represents days of blood collection). **e** Level of IL-6, IL-10 and IFN-γ cytokines in sera of immunized mice at 35th day of primary immunization quantified using cytokine ELISA (normalized with control) (one-way ANOVA, Tukey’s Multiple comparisons test; for IL-6 ***p < 0.001 and ****p < 0.0001 indicate statistically significant difference between the respective groups; for IL-10, **p < 0.01 and ***p = 0.0004 indicate statistically significant difference between the respective groups; for IFN-ɣ, *p < 0.05, **p < 0.01 and ***p = 0.0001 indicate statistically significant difference between the respective groups; ns indicates non-significance)

To evaluate immunogenicity of the nanovaccines, 4–6 week old BALB/c mice (n = 14) were intranasally immunized (Fig. 6a). Immunoblots with 28th day immunized sera showed that both Sd1 (source of encapsulants) and Sf2a (most common strain in circulation) could be recognized by the IpaC antibodies present in sera of the vaccinated animals suggesting the possibility of cross-protection (Fig. 6b). All immunized animals showed high serum IgG and IgA antibody response (except for the group immunized with free form of the encapsulants which showed a slightly lower IgG response) at all three time points compared to the control/blank NPs (Fig. 6c and d, plates coated with whole cell lysate). Cytokine response was determined at day 35 of primary immunization (Fig. 6e). The group immunized with the free encapsulants showed high IL-6 and IL-10 response.

NV1 showed relatively higher IL-6 response (pro-inflammatory cytokine that helps in the maturation of antibody-secreting cells) [77] compared to NV2 and 3. The difference in IL-10 response between NV1 and 2 as well as between the free group and NV3 was not significant. IL-10 response of NV3 was also higher when compared to NV1 and 2. Elevation of IL-10 levels shown by all the three nanovaccines is expected to stimulate B cell proliferation, improve antibody secretion and limit inflammatory responses [78]. The IFN-Gamma level, representative of Th1 response, was highest in NV3 (containing dual immunostimulants) compared to NV2 and the free encapsulants which had single immunostimulants each (CpG DNA and LPS respectively). A higher Th1 response is known to be beneficial in eradication of intracellular pathogens including *Shigella* [79]. Taken together, all the nanovaccines showed significant antibody and cytokine response and hence, were considered to be significantly immunogenic.

### Percentage survival against *Shigella* challenge

**Fig. 7:**
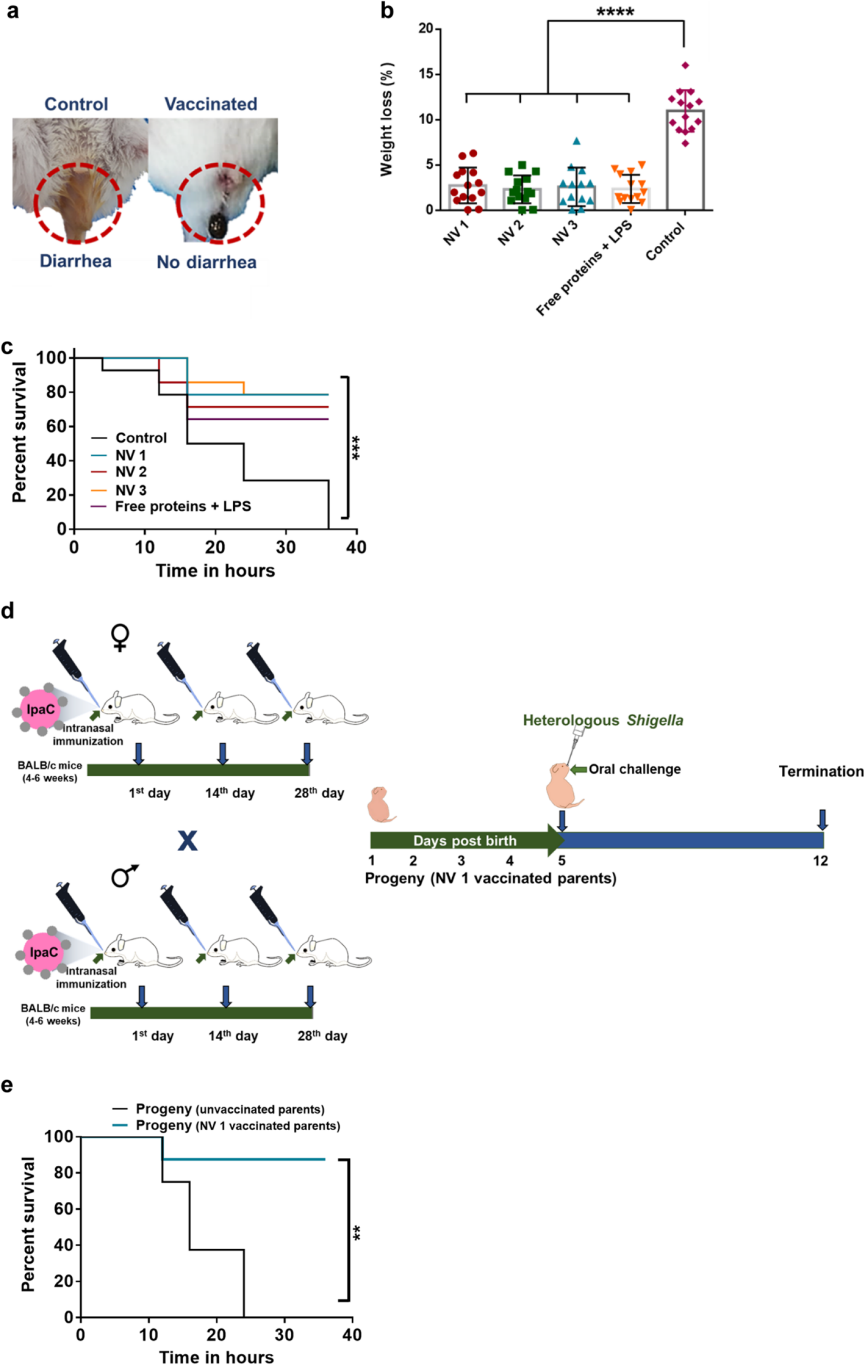
*Shigella* challenge. **a** Visible diarrhea was observed in control/blank NP group (left image) and fecal pellet was observed in all immunized groups (right, representative image), 4 h post intraperitoneal challenge with heterologous *Shigella*. **b** Percentage loss of weight in the treated groups 12 h post challenge (Kruskal–Wallis test, Dunn’s multiple comparisons posttest, ****p < 0.0001 indicates statistically significant difference of the treated groups with respect to control). **c** Percentage survival of the treated groups against intraperitoneal challenge with heterologous *S. flexneri* 2a, 1 × 10^9^ CFU (Log Rank test ***p = 0.0001 indicates statistically significant difference with respect to control) (n = 14). **d**, **e** Passive immunity analysis—mice immunized with NV1 were bred to produce progeny after 3rd dose of immunization (28 days) and the obtained 5 day old neonates were orally challenged with *S. flexneri* 2a, 5 × 10^8^ CFU (Log Rank test **p = 0.0075 indicates statistically significant difference with respect to control) (n = 8)

To assess the protective ability of the nanovaccines, all immunized animals were intraperitoneally challenged on the 56th day with a high dose of heterologous multi-drug resistant *S. flexneri* 2a (1 × 10^9^ CFU/mouse) (Additional file 1: Fig. S6) [26, 80]. The animals were closely monitored for symptoms such as diarrhea, lethargy, ruffling of fur and weight loss till 14 days of challenge (Additional file 1: Table S1). Visible diarrhea was observed in the control group 4 h post challenge (Fig. 7a), while all the vaccinated groups were free of diarrhea showing fecal pellets. Interestingly, the percentage loss of weight 12 h post challenge was minimal for all the vaccinated groups, whereas, significant weight loss was observed in the control group (Fig. 7b). All vaccinated animals recovered the loss in weight within 14 days of challenge (Additional file 1: Fig. S7). Interestingly, groups immunized with NV1 or NV3 showed three deaths each (78.6% survival) (Fig. 7c), whereas, the group immunized with NV2 showed an additional death (71.4% survival). The group immunized with free stabilized IpaC, IpaB^44-310^ and LPS showed 5 deaths (64.3% survival). All animals in the control group died before 40 h of challenge while all the survivors were free of diarrhea, lethargy and other disease symptoms till termination of the study (70th day). Therefore, based on these observations, all the developed vaccines were considered to be cross-protective in nature and hence, show potential for translational development. Further, formulation of stabilized IpaC into a nanovaccine increased its protective ability when compared to free protein along with IpaB^44-310^ and LPS. Additionally, as NV1 (involving a single stabilized antigen) resulted in significant protection, it shows potential for facile formulation of a minimalist vaccine.

### Passive immunity in neonatal mice

As *Shigella* leads to greater mortality in infants, the prospect of passive immunization was explored. For this, in a preliminary study, mice immunized with NV1, the simplest group containing a single antigen (as it could show survival equivalent to NV3 with dual antigens and immunostimulants, Fig. 7c) were bred and the obtained progeny (5 day old) were challenged with heterologous Sf2a orally (5 × 10^8^ CFU) (Fig. 7d). The percentage survival in the group with vaccinated parents was 87.5% with only one death out of 8 neonates, whereas, the control neonates died within 24 h of challenge (Fig. 7e). This demonstrated the potential of the nanovaccine to provide passive protection.

Although considerable research has been undertaken around the globe to obtain a safe and protective *Shigella* vaccine, a commercial vaccine is still not available. Glyco-conjugate vaccine candidates have been popular for *Shigella* vaccine research as LPS have been found responsible in providing protection against reinfection with the same strain of *Shigella* [81]. However, as there are more than 50 serotypes and sub-serotypes of *Shigella*, a glyco-conjugate vaccine is unable to provide protection against an infection caused by a non-parent type of bacteria. Therefore, sub-unit vaccines based on conserved proteins found in all strains of *Shigella* are increasingly explored to obtain ‘cross-protection’ [23, 24, 78]. Another common strategy is the usage of the outer membrane vesicles such as the relatively newer generalized module for membrane antigens (GMMA) obtained from over-vesiculating strains with mutations in the LPS genes to reduce toxicity [82–85]. These require large scale culture of *Shigella*. This can be mitigated by using immunogenic conserved recombinant antigens obtained by expressing them in non-pathogenic *E. coli*. Further, involving nanoparticulate systems can significantly increase stability of the antigens by protecting them from degradation and increasing their circulation time leading to relatively higher immunogenicity. This can contribute towards a pan-*Shigella* vaccine which can provide effective cross-protection against all strains of *Shigella* including *S. dysenteriae* 1, the causative organism of the most severe form of the disease. It is in this light that the current study holds significance.

## Conclusion

As our objective was to obtain vaccines applicable for different settings (lower/low/middle/high income countries), we explored surface-modified biomimetic nanovaccines. We describe a facile platform technology for rapid development of non-invasive, biomimetic, cross-protective PLGA nanovaccines for shigellosis. The nanovaccines (with increasing level of complexity- NV1, 2 and 3) had conserved recombinant protein(s)/immunostimulant of Sd1 origin as encapsulant(s) and were surface modified with CpG DNA (pathogen associated-molecular pattern, found in bacterial genome) and/or IpaC (employed by *Shigella* to enter host cells), resulting in enhanced cellular uptake and significant cross-protection against multi-drug resistant *Shigella*. Interestingly, the group immunized with NV1 (single antigen), with a survival of ~ 80%, also resulted in passive protection in 5 day-old neonates suggesting that parental immunization can provide protection in infants, the most vulnerable population in context of shigellosis. Therefore, if the goal is to formulate a minimalist vaccine for mass immunization, especially in the developing world where cost could be a consideration, NV1 could be the preferred choice. However, if resources are not a constraint, NV2 or 3, with higher Th1 response, could be explored.

## Supplementary Information

**Additional file 1. **Nanoparticle formulation, quantification of LPS, loading efficiency with more than one encapsulant, adsorption efficiency of IpaC on surface of NPs, characterization of CpG DNA modified NPs, antibiogram, assessment of clinical score and recovery of weight loss in challenged mice.

## Data Availability

All data generated or analyzed during this study are included in this published article and its additional file.

## Abbreviations

PLGA: Poly (lactide-*co*-glycolide)
Ipa: Invasion plasmid antigen
LPS: Lipopolysaccharide
NPs: Nanoparticles
NVs: Nanovaccines
MALT: Mucosal-associated lymphoid tissues
PAMP: Pathogen-associated molecular pattern
T3SS: Type III secretion system
LB: Luria Bertani
Sf2a: *Shigella flexneri* 2a
Sd1: *Shigella dysenteriae* 1
TBA: Thiobarbituric acid assay
SDS-PAGE: Sodium dodecyl sulfate–polyacrylamide gel electrophoresis
DCM: Dichloromethane
PVA: Polyvinyl alcohol
TRITC: Tetramethylrhodamine isothiocyanate
EDC: *N*-(3-Dimethylaminopropyl)-N′-ethylcarbodiimide hydrochloride
NHS: *N*-Hydroxysuccinimide
LDH: Lactate dehydrogenase
DMEM: Dulbecco’s Modified Eagle’s medium
HRP: Horseradish peroxidase
ELISA: Enzyme-linked immunosorbent assay
IL: Interleukin
IFN: Interferon
