# Supplementary material for: Facile synthesis of multi-faceted, biomimetic and cross-protective nanoparticle-based vaccines for drug-resistant Shigella: a flexible platform technology
Source: J Nanobiotechnology. 2023 Jan 29;21:34. doi: 10.1186/s12951-023-01780-y (PMC9884485; doi:10.1186/s12951-023-01780-y)
Supplement: Supplementary file 1 — Additional file 1. Nanoparticle formulation, quantification of LPS, loading efficiency with more than one encapsulant, adsorption efficiency of IpaC on surface of NPs, characterization of CpG DNA modified NPs, antibiogram, assessment of clinical score and recovery of weight loss in challenged mice. [file 12951_2023_1780_MOESM1_ESM.pdf]

Additional file 1

Title: Facile synthesis of multi-faceted, biomimetic and cross-protective nanoparticle-based vaccines for drug-resistant *Shigella*: A flexible platform technology

Authors: Namrata Baruah[a,b], Nadim Ahamad[a], Prolay Halder[c], Hemanta Koley[c] and Dhirendra S. Katti[a,b,*]

[a]Department of Biological Sciences and Bioengineering, Indian Institute of Technology Kanpur; Uttar Pradesh, 208016, India.

[b]The Mehta Family Centre for Engineering in Medicine, Indian Institute of Technology Kanpur; Uttar Pradesh, 208016, India.

[c]Division of Bacteriology, ICMR-National Institute of Cholera and Enteric Diseases; Kolkata, West Bengal, 700010, India.

*Dhirendra S. Katti

Email: dsk@iitk.ac.in

Table of Contents

Figures

S1. Double Emulsion Solvent Evaporation Method of nanoparticle fabrication……………......3

S2. Quantification of LPS. …………………………………………………………........3

S3. Comparison of loading efficiency of model antigen BSA…………………………………..4

S4. Physical adsorption efficiency of IpaC…………………………………………….......5

S5. Size and Zeta potential of CpG DNA modified NPs………………………………..........5

S6. Antibiogram ………………………………………………………………………...6

S7. Recovery of weight loss in challenged mice…………………………………..…………8

Table

Table S1. Clinical score of challenge study. ………………………………………………….....7

Figures

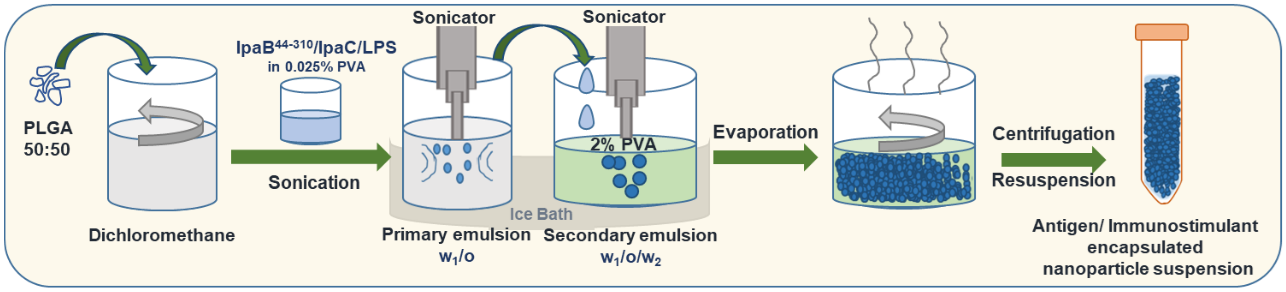

Figure S1. Double emulsion solvent evaporation method of nanoparticle fabrication. PLGA 50:50 is dissolved in dichloromethane to make a 2.5% solution. After addition of antigen/ immunostimulant solution in 0.025% PVA (polyvinyl alcohol) to the PLGA solution, the mixture is sonicated to obtain a primary emulsion which is added dropwise into 2% PVA and re-sonicated to obtain a secondary emulsion. After evaporation of the organic solvent dichloromethane, the nanoparticle suspension is centrifuged to obtain the NP pellet which is washed multiple times to remove the residual PVA and resuspended in ultra-pure water for lyophilization.

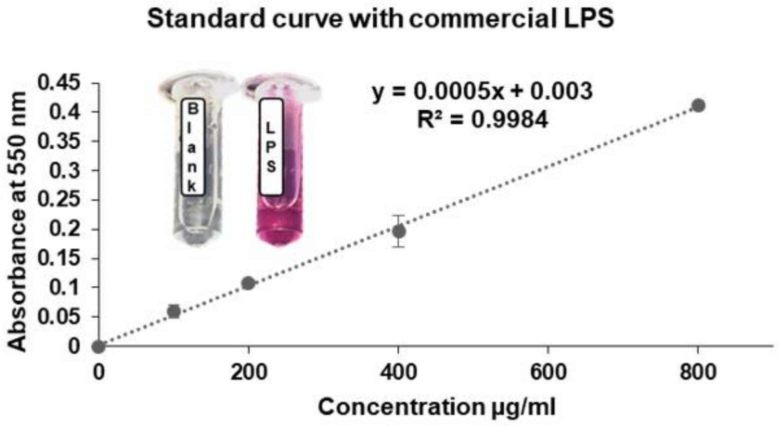

Figure S2. Quantification of LPS- Thiobarbituric acid assay of extracted *S. dysenteriae* 1 LPS. Presence of KDO (2-keto-3-deoxy-octonate) results in pink coloration which was quantified using

a standard curve which was obtained with commercial *Shigella* LPS (procured when it was commercially available).

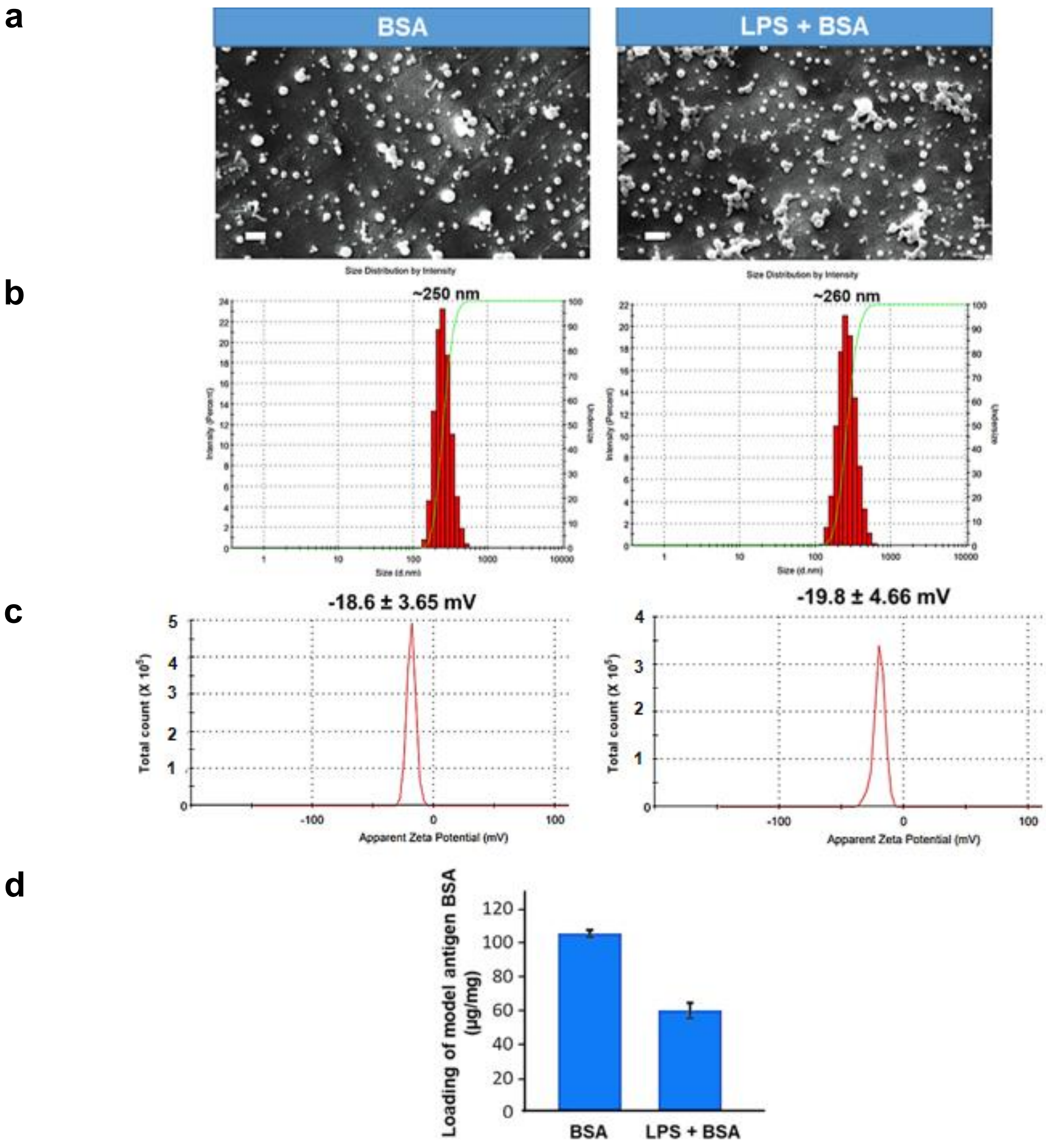

Figure S3. Simultaneous encapsulation of the model antigen BSA along with the immunostimulant LPS decreases loading of BSA in the NPs. **a** Scanning electron micrographs (scale bar- 500 nm) and (**b** and **c**) hydrodynamic size and zeta potential of BSA loaded and LPS + BSA loaded NPs. **d** Amount of BSA loaded in µg per mg of NPs.

a

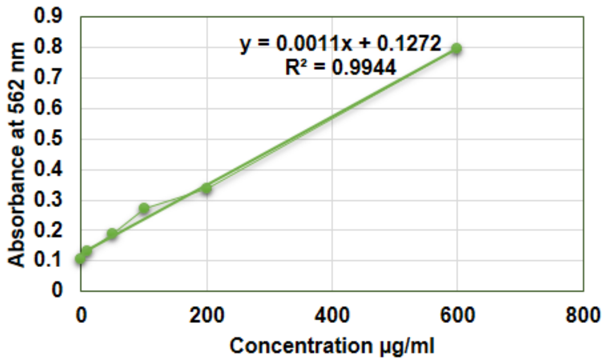

b

Sample type	IpaC	IpaC + CpG
Loaded	265 µg/ml	265 µg/ml
Supernatant	136 ± 3 µg/ml	146 ± 2 µg/ml
Actual amount of protein in 5 mg NP	127 ± 3 µg/ml (~49%)	119 ± 2 µg/ml (~45%)

Figure S4. Physical adsorption efficiency of IpaC on the surface of unmodified and CpG DNA modified NPs, an indirect quantification using BCA assay. **a** Standard curve used to determine the adsorption efficiency. **b** Calculated adsorption of IpaC on unmodified and CpG DNA modified NPs.

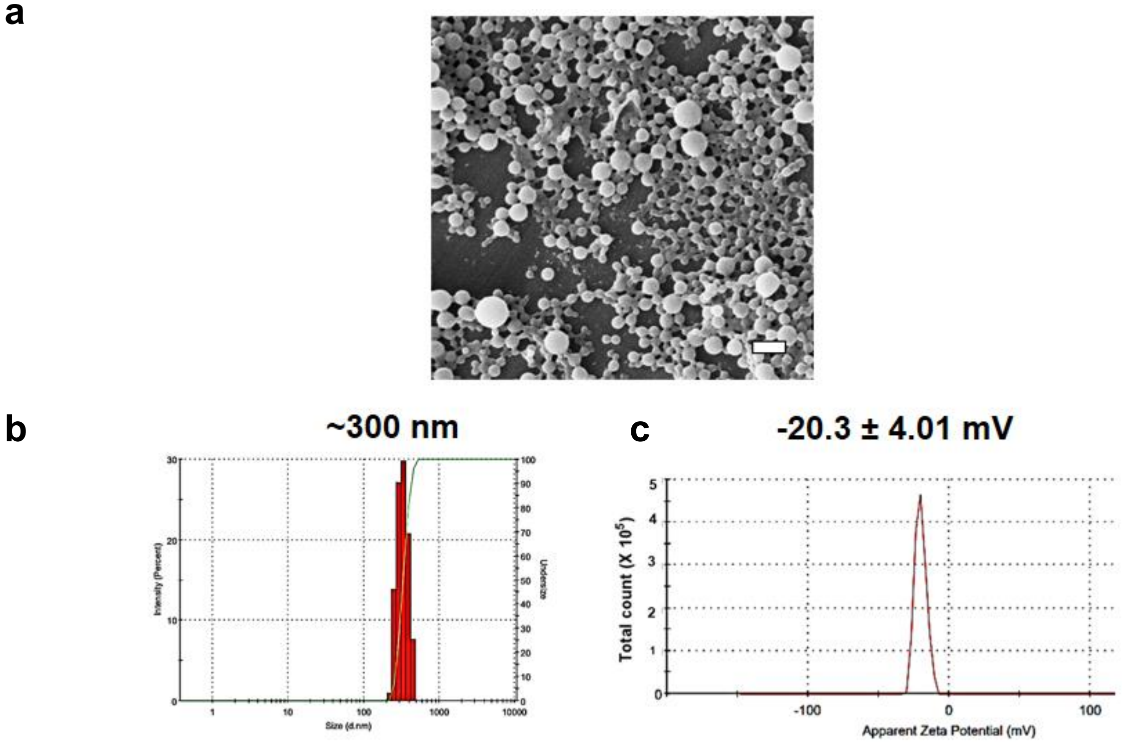

Figure S5. Characterization of CpG DNA modified PLGA NP. **a** Scanning electron micrograph (scale bar- 1 µm), **b** Hydrodynamic size and **c** Zeta potential.

a

Antibiotic	Zone (mm)	Comments
Cefotaxime	12	Resistant
Ceftriaxone	23	Sensitive
Imipenem	12	Resistant
Ciprofloxacin	11	Resistant
Neomycin	14	Intermediate
Cefixime	10	Resistant
Trimethoprim/ Sulfamethoxazole	<10	Resistant
Lomefloxacin	<10	Resistant
Co-trimoxazole	<10	Resistant
Nalidixic Acid	<10	Resistant
Tetracyclin	<10	Resistant
Streptomycin	<10	Resistant
Methicillin	<10	Resistant
Chloramphenicol	<10	Resistant
Ampicillin	<10	Resistant
Norfloxacin	<10	Resistant
Clarithromycin	<10	Resistant
Erythromycin	<10	Resistant
Kanamycin	11	Resistant

b

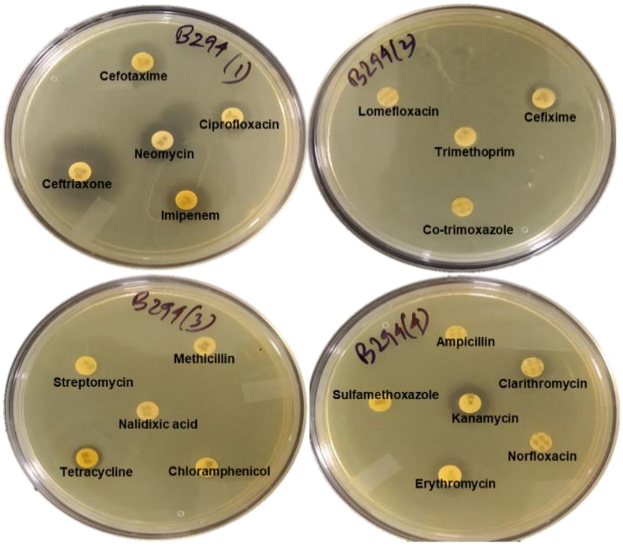

Figure S6. Antimicrobial susceptibility of *Shigella flexneri* 2a by disc diffusion method. **a** Table of results. **b** Antibiogram. The bacteria was found to be resistant against all tested antibiotics except for Ceftriaxone.

Table S1: Clinical score of challenge study.

Group	Parameter studied	Observation	Duration of study
**PBS-immunized Non-infected Control**	Ruffled Fur	**No symptoms**	**14 days post** heterologous challenge
	Weight Loss	**No symptoms**	
	Diarrhea	**No symptoms**	
	Lethargy	**No symptoms**	
	Survival	**100%**	
**Non-immunized infected**	Ruffled Fur	**Severe**	**40 hours post** heterologous challenge
	Weight Loss	**Severe**	
	Diarrhea	**Severe diarrhea within 4 hours**	
	Lethargy	**Severe**	
	Survival	**0%**	
**NV1 immunized infected**	Ruffled Fur	**No symptoms**	**14 days post** heterologous challenge
	Weight Loss	**Not significant**	
	Diarrhea	**No symptoms**	
	Lethargy	**No symptoms**	
	Survival	**78.6%**	
**NV2 immunized infected**	Ruffled Fur	**No symptoms**	**14 days post** heterologous challenge
	Weight Loss	**Not significant**	
	Diarrhea	**No symptoms**	
	Lethargy	**No symptoms**	
	Survival	**~71.4%**	
**NV3 immunized infected**	Ruffled Fur	**No symptoms**	**14 days post** heterologous challenge
	Weight Loss	**Not significant**	
	Diarrhea	**No symptoms**	
	Lethargy	**No symptoms**	
	Survival	**78.6%**	

**Free protein + LPS immunized infected**	Ruffled Fur	**No symptoms**	**14 days post** heterologous challenge
	Weight Loss	**Not significant**	
	Diarrhea	**No symptoms**	
	Lethargy	**mild**; recovered over time	
	Survival	**64.3%**	

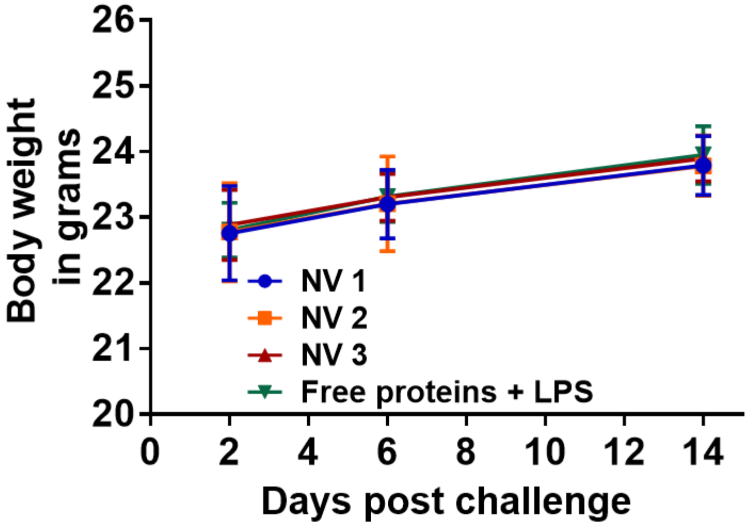

Figure S7. Change in body weight of vaccinated mice post challenge. All vaccinated animals recovered the loss in weight within 14 days of challenge.
